# Unravelling the Complex Genetics of Karnal Bunt (*Tilletia indica*) Resistance in Common Wheat (*Triticum aestivum*) by Genetic Linkage and Genome-Wide Association Analyses

**DOI:** 10.1534/g3.119.400103

**Published:** 2019-03-01

**Authors:** Livinus Emebiri, Sukhwinder Singh, Mui-Keng Tan, Pawan K. Singh, Guillermo Fuentes-Dávila, Francis Ogbonnaya

**Affiliations:** * NSW Department of Primary Industries, Southern Cropping Systems, Private Mail Bag, Wagga Wagga, NSW, 2650, Australia; † Graham Centre for Agricultural Innovation (NSW Department of Primary Industries and Charles Sturt University), Wagga Wagga, NSW 2650, Australia; ‡ International Maize and Wheat Improvement Center (CIMMYT), Apdo 6-641, 06600, Mexico D.F., Mexico; § NSW Department of Primary Industries, Woodbridge Rd, Menangle NSW 2568, Australia; ** INIFAP-CIRNO, Campo Experimental Norman E. Borlaug, Apdo. Postal 155, Km 12 Norman E. Borlaug, Cd. Obregon, Sonora, CP 85000, Mexico CP 85000; †† Grains Research and Development Corporation, P.O. Box 5367, Kingston, ACT 2604, Australia

**Keywords:** Karnal bunt resistance, wheat (*Triticum aestivum*), quantitative trait locus (QTL), genome-wide association, single-nucleotide polymorphism, Genetics of Immunity

## Abstract

Karnal bunt caused by *Tilletia indica* Mitra [syn. *Neovossia indica* (Mitra) Mundkur] is a significant biosecurity concern for wheat-exporting countries that are free of the disease. It is a seed-, soil-and air-borne disease with no effective chemical control measures. The current study used data from multi-year field experiments of two bi-parental populations and a genome-wide association (GWA) mapping panel to unravel the genetic basis for resistance in common wheat. Broad-sense heritability for Karnal bunt resistance in the populations varied from 0.52 in the WH542×HD29 population, to 0.61 in the WH542×W485 cross and 0.71 in a GWAS panel. Quantitative trait locus (QTL) analysis with seven years of phenotypic data identified a major locus on chromosome 3B (R^2^ = 27.8%) and a minor locus on chromosome 1A (R^2^ = 12.2%), in the WH542×HD29 population, with both parents contributing the high-value alleles. A major locus (R^2^ = 27.8%) and seven minor loci (R^2^ = 4.4–15.8%) were detected in the WH542×W485 population. GWA mapping validated QTL regions in the bi-parent populations, but also identified novel loci not previously associated with Karnal bunt resistance. Meta-QTL analysis aligned the results from this study with those reported in wheat over the last two decades. Two major clusters were detected, the first on chromosome 4B, which clustered with *Qkb.ksu-4B*, *QKb.cimmyt-4BL*, *Qkb.cim-4BL*, and the second on chromosome 3B, which clustered with *Qkb.cnl-3B*, *QKb.cimmyt-3BS* and *Qkb.cim-3BS1*. The results provide definitive chromosomal assignments for QTL/genes controlling Karnal bunt resistance in common wheat, and will be useful in pre-emptive breeding against the pathogen in wheat-producing areas that are free of the disease.

Karnal bunt is a disease of wheat (*Triticum aestivum* L.) caused by the hemibiotrophic fungus *Tilletia indica* Mitra [syn. *Neovossia indica* (Mitra) Mundkur]. The disease is of little agronomic significance (Bonde *et al.* 1997), but it is the subject of strict quarantine control by several wheat-importing countries (Beattie & Biggerstaff 1999), and therefore a major biosecurity concern for wheat-exporting countries that are free of the disease. Karnal bunt is established in parts of Asia (Afghanistan, Iraq, Nepal, Pakistan, Iran) (Warham 1986; Torabi *et al.* 1996), and several non-Asian countries, including Mexico (Duran 1972), the southern part of the Rio Grande do Sul in Brazil (Da Luz *et al.* 1993), the USA (Ykema *et al.* 1996) and the northern Cape Province of South Africa (Crous *et al.* 2001). It is not present in Australia, but the weather across the wheat-producing regions would be suitable for the infection of susceptible varieties (Murray and Brennan 1998). Should the pathogen become established across all of Australia’s wheat-growing regions, and Australian wheat is subject to quarantine restrictions in foreign markets, the economic impact would amount to $A1.5 billion per annum (Wittwer *et al.* 2005).

Karnal bunt infections occur late during development of the host plant, and because the pathogen is seed-soil-and airborne, successful chemical control of the disease is limited and not cost-effective. Once established, it is extremely difficult to eradicate as the teliospores can remain viable in soil for several years, while stubble burning only serves to disseminate the spores over long distances (Villareal *et al.* 1995). Improving host resistance is an important pre-emptive measure to prevent disease establishment (Brooks *et al.* 2006), but resistance is limited in commercially cultivated wheat varieties, and as such, progress in breeding has remained modest. The disease is complex, highly dependent on the environment, and the method for phenotyping is too tedious and labor-intensive for testing segregating populations (Sukhwinder-Singh *et al.* 2003; Singh *et al.* 2007; Brooks *et al.* 2006). The development of molecular markers closely linked to resistance genes is a feasible approach that would improve selection efficiency, and has the potential to improve selection of resistant genotypes even in the absence of the pathogens (Sukhwinder-Singh *et al.* 2012).

Karnal bunt resistance in common wheat is mediated by multiple genes. Fuentes-Davila *et al.* (1995) suggested six genes, designated Kb1, Kb2, Kb3, Kb4, Kb5, and Kb6, while Villareal *et al.* (1995) postulated a minimum of three genes for resistance. Efforts have been made to identify chromosomal location of the genes controlling Karnal bunt resistance in common wheat using the quantitative trait locus (QTL) mapping approach (Nelson *et al.* 1998; Sukhwinder-Singh *et al.* 2003, 2012; Singh *et al.* 2007; Kumar *et al.* 2007; Kumar *et al.* 2015; Kaur *et al.* 2016). However, these studies were based on a small number of restriction fragment length polymorphisms (RFLP) and PCR-based simple sequence repeats (SSRs). With advances in DNA sequencing and genotyping technologies, the cost of genotyping has decreased and thousands of single-nucleotide polymorphisms (SNP) can now be mapped to chromosomal locations in the wheat genome (Cavanagh *et al.* 2013; Wang *et al.* 2014). SNPs are single-point mutations of DNA in which one nucleotide in a particular locus is substituted with another one (Simko *et al.* 2012), and since the exact nature of the allelic variation can be determined from the sequence information, the annotated, whole-genome sequence of cereals and other additional resources offer new opportunities to explore the genetic basis and putative gene or genes associated with Karnal bunt resistance.

The aim of this study was to identify new quantitative trait loci for Karnal bunt resistance in common wheat by using linkage mapping complemented with association mapping. When conducted together, linkage and association mapping approaches provide a powerful method for QTL detection and the identification of molecular markers amenable to immediate deployment in breeding. In combination with high-throughput iSelect 90K SNP marker assays, we sought to query the genetic basis underlying resistance to Karnal bunt infection in common wheat.

## Materials and methods

### Plant materials

### Recombinant inbred line populations

Two bi-parental populations of wheat recombinant inbred lines (RILs) were used for this study. The first population was derived from a cross between WH542 and HD29, while the second was derived from the WH542×W485 cross, with HD29 and W485 as the resistant parents, respectively. The resistant lines, HD29 (HD2160-HD1977/HD7449-HD1944/HD2136) and W485 (WL923/HD2160/UP368), share a common parent (Sharma *et al.* 2005) and are probably the most rigorously studied sources of resistance to the fungus *T. indica*. WH542 is a highly susceptible cultivar derived from the widely adapted breeding line Kauz (Jupateco/Bluejay//Ures) (Fuentes-Davila and Rajaram 1994; Nagarajan *et al.* 1997; Chhuneja *et al.* 2008; Kumar *et al.* 2007; Kumar *et al.* 2015; Kaur *et al.* 2016).

### Association mapping panel

For association mapping, we screened one hundred and nineteen wheat genotypes, drawn largely from varieties of significance to Australia’s wheat industry and parents used in breeding programs by the major breeding companies (Emebiri *et al.* 2019). In addition, 25 accessions from the CIMMYT wheat germplasm bank were also tested, and these include the genotype Munal #1 used as the resistant check, and the highly susceptible Indian wheat variety WL-711 used as a comparative control.

### Screening for Karnal bunt resistance

Field experiments for disease screening were carried out during the cropping seasons of 2001, 2002, 2003, 2004 and 2005 at Ludhiana India (Sukhwinder-Singh *et al.* 2012) and in 2014, 2015 and 2016 at the Norman E. Borlaug Experimental Station of the International Maize and Wheat Improvement Center (CIMMYT), Obregon, located in the Yaqui valley, on the northwest coast of mainland Mexico in the state of Sonora (27.4828° N, 109.9304° W). The genotypes were sown in un-replicated plots consisting of double rows measuring 1-m in length on 80 cm wide raised beds, with resistant control repeated twice and susceptible control replicated three times.

At the boot stage (growth stage GS47, Zadoks *et al.* 1974), five heads of each experimental line were artificially inoculated by injecting an inoculum suspension (1ml) into the leaf sheaths with a hypodermic syringe (Aujla *et al.* 1982). To prepare the inoculum, Karnal bunt infected wheat kernels were mixed with Tween 20 solution in a glass tube, which was shaken and then filtered with 60 μm mesh and allowed to stand for 24 hr. The collected teliospores were placed in 0.6% sodium hypochlorite for 2 min and centrifuged at 3,000 rpm for 5 sec. The supernatant was discarded and teliospores were rinsed with distilled water, then the solution was centrifuged for 5 sec at 3,000 rpm. The rinse and centrifuge steps were repeated. The teliospores were transferred to 2% water agar under sterile condition and incubated at 18-22° until germination was detected. Pieces of water agar with germinating teliospores were placed inversely onto Petri dishes with potato-dextrose-agar (PDA) to stimulate production of secondary sporidia. Nine days later, the Petri dishes were flooded with sterile water and scraped with a sterile spatula, and the suspension transferred to new Petri dishes with PDA for colony growth. Once the Petri dishes were covered with fungal colonies, the agar was cut into pieces and put inversely on sterile glass petri dishes, into which distilled water was added and secondary sporidia were harvested. Inoculum concentration was adjusted to 10,000 sporidia per millilitre, using a hemocytometer (Fuentes-Davila and Rajaram 1994).

Stems of the inoculated heads were identified with a piece of red plastic. Appropriate humidity was created in the field via intermittent misting with overhead sprinklers during the inoculation period. Plants were misted for 20 min, five times are day. At maturity, the five inoculated ears were harvested and threshed separately, and the number of infected and uninfected seeds per ear was counted. The disease resistance was quantified as the percentage of infected grains in each ear (Fuentes-Davila and Rajaram 1994).

### SNP genotyping and linkage map construction

Genomic DNA was extracted from leaf tissue ground in liquid nitrogen, using the CTAB-DNA method as described by Singh *et al.* (2007). SNP genotyping was performed as a service by the Department of Primary Industries (Victorian AgriBiosciences Center, Bundoora, VIC3083, Australia), which used the Illumina Infinium iSelect 90K SNP wheat array (Wang *et al.* 2014).

For each RIL population, SNP markers were filtered to remove those with distorted segregation and/or allele frequencies of less than 0.4. Linkage map construction was carried out using the minimum spanning tree (MST) method (Wu *et al.* 2008), as implemented in the R package ASMap (Taylor and Butler 2017). First, the markers were imported into R/ASMap to perform initial marker ordering by running the ‘mstmap.data.frame’ command, with p.value = 1E-12, and noMap.dist = 30. Further improvements on the genetic maps were made by checking genotype phase of the markers. This was accomplished using MultiQTL (http://www.multiqtl.com/) to detect intervals with >0.5 recombination rate, and correct these by changing phase of the affected group of markers. Following this step, the markers were again run through R/ASMap to obtain the final order. In cases where this resulted in split chromosomes, the groups were joined using the ‘mergeCross’ command to obtain a single chromosome group. Missing values were imputed using the rules of Martínez and Curnow (1992), and then the wheat consensus map (Wang *et al.* 2014) was used to orient mapped markers with respect to the short and long arms of the respective chromosomes.

### Statistical analysis

All the phenotypes were positively skewed (Supplementary Material Figure 1S) and could not be transformed to normality, so the data were analyzed on their original scale. The GGE biplot methodology (Yan and Tinker 2005) was used to examine the relationships among years, genotypes and the genotype-by-year interactions (G×E interaction). The R package ‘GGEBiplotGUI’ (Frutos *et al.* 2014) was used to analyze a two-way genotype-environment dataset for each population. The data were environment-centered (centering = 2) without any scaling, and subjected to the environment-metric preserving singular value decomposition (SVP = 2). The results were visualized in a biplot of the first two axes scores, and interpreted according to Yan and Tinker (2006).

The broad-sense Heritability (*h*^2^) for each population was estimated using the R package, ‘heritability’ (Kruijer *et al.* 2015). The Whole Genome Average Interval Mapping (WGAIM) approach (Verbyla *et al.* 2012) was used to perform QTL analysis across phenotyping years. The analysis was carried out in R in four steps. First, a base model was fitted to the phenotypic data using ASReml-R (Butler *et al.* 2009), which treated environments like a replication in the following format:

$$y=X\beta +\text{Z}u+{\text{Z}}_{g}g+e$$

where y is a vector of trait observations, Xβ is the fixed component that captures the mean effects of parents and the RIL lines, Ζu is a random environment component, ${\text{Ζ}}_{g}g$ was used to model the underlying genetic variation of the trait among the RIL lines and e is the residual error component.

In the second step, the genetic data were imported using the ‘read.cross’ command of R/QTL (Broman *et al.* 2003), and converted into an ‘interval’ object using the ‘cross2int’ command of WGAIM package (Taylor and Verbyla 2011), with co-located markers placed in consensus bins. Then, the genetic ‘interval’ data were merged with the base model phenotypic data, and in the final step, WGAIM extended the model by incorporating all markers simultaneously as random covariates to detect main effect QTL. This method has been shown to outperform the standard composite interval mapping approach (Verbyla *et al.* 2007). Detailed summaries of significant QTL, including their position, effect size, significance and contribution to the overall genetic variance were provided by the package. The genome-wide significance level for declaring a significant QTL was established by the Li and Ji (2005) method at α = 0.05, which returned a value of 3.1 for the WH542×HD29 population, and 3.0 for the WH542×W485 population.

### Genome-wide association analysis

Phenotypic data from each of three years (2014, 2015, and 2016) were pooled for association analysis. Genome-wide association (GWA) analysis was based on the mixed model described by Yu *et al.* (2006). The analysis was conducted with the univariate multi-locus mixed model (MLMM) procedure (Segura *et al.* 2012), which uses a stepwise regression method to sequentially incorporate significant markers as covariates in the GWA model before scanning all other markers. Compared with traditional single-locus approaches, this method is reported to increase the detection power and reduce the level of false discovery (Sauvage *et al.* 2014). A kinship matrix was used to control the rate of false positives, and from the optimal GWA model (based on mbonf, the multiple Bonferroni criterion), markers exhibiting a Bonferroni *P*-value < 0.05 were identified as significant. The proportion of the total genetic variance explained by significant loci was determined by dividing the sum of squares for each marker by the total corrected model sum of squares, in an ANOVA model where population structure and environment were sequentially added as covariates.

### Meta-QTL analysis

To identify meta-QTL, we first projected results from this study and those previously reported onto the POPSEQ genetic map (Chapman *et al.* 2015). QTL previously reported by various authors include *Qkb.cnl-3B* detected by Nelson *et al.* 1998 on chromosome 3B, and the *QKB.ksu-4BL* locus detected by Sukhwinder-Singh *et al.* (2003, 2012); Singh *et al.* (2007). Sehgal *et al.* (2008), Kumar *et al.* (2007; 2015) and Kaur *et al.* (2016) also reported marker-trait associations. More recently, Brar *et al.* (2018) identified QTL for Karnal bunt resistance on chromosome 3B (*Qkb.cim-3BS*) and chromosome 4B (*Qkb.cim-4BL*) using the genotype-by-sequencing approach. With the exception of Brar *et al.* (2018), all the previous reports were based on microsatellite (SSR) markers. We used the wheat consensus map of Maccaferri *et al.* (2015) to identify nearby, single-nucleotide polymorphic markers closely linked to the SSRs. For this purpose, the QTL region was assumed to extend ±15 cM flanking the locus, resulting in a search interval of 30 cM. This is equivalent to the maximum recombination fraction of θ = 0.3 generally used for genetic linkage mapping (Botstein *et al.* 1980). Meta-QTL analyses were performed using the functions of Goffinet and Gerber (2000) as implemented in the BioMercator software (Sosnowski *et al.* 2012).

### Data availability

Supplementary File 1S is a summary of genetic linkage map and phenotypic distribution of percent Karnal bunt infected grains in two wheat recombinant inbred line populations, and a genome-wide association mapping panel of commercial varieties. Files S2 and S3 are plots of allele effects of QTL detected for Karnal bunt resistance in the WH542×HD29 and WH542×W485 populations. Phenotypic and genotypic data for all populations are also accessible through the CIMMYT-Australia-ICARDA Germplasm Evaluation (CAIGE) suite of projects (http://caigeproject.org.au/). Supplemental material available at Figshare: https://doi.org/10.25387/g3.7374200.

## Results

### Genetic linkage maps

A total of 8403 polymorphic SNP markers were generated in the WH542×HD29 population using the Illumina platform, with a mean call rate of 94.0%. In comparison, the call rate among parental DNA ranged from 43.0% (HD29) to 44.0% (WH542). Visual inspection of clustering in GenomeStudio showed that the parents clustered very differently compared to the progeny, suggesting that the parental DNA used for genotyping might not be from the correct parents. The consequence is that a high proportion of the SNPs could not be correctly phased, but following linkage map construction as described above, 1535 SNPs were mapped to chromosome locations on the A, B and D genomes of wheat (Supplementary Material Table 1S) in the WH542×HD29 population. The mapped markers, on average, satisfied the expected ratio of 1:1 (AA = 50.1%; BB = 49.9%), with equal allele frequencies across the whole genome. The total length of the genetic linkage map was 6548.4 cM, with an average marker interval of 4.5 cM. Chromosome 2B had the highest number of mapped markers, with an average interval of 2.6 cM, while chromosome 4D had the lowest coverage (Supplementary Material Table 1S).

In the WH542×W485 population, 13764 SNPs were polymorphic with a mean call rate of 94.9%. The parents (WH542 and W485) were also observed to have low call rates (53.0% and 48.0%, respectively), and to cluster differently compared to the progeny. Of the total number of polymorphic markers, 1628 SNP markers were placed on the linkage map, covering a length of 3745.8 cM. The markers satisfied the expected ratio of 1:1 segregation, with 49.0% of ‘AA’ alleles, and 51.0% of the ‘BB’ alleles. The average marker interval was 2.3 cM, with 99.2% of the genome containing markers spaced at within 20 cM to the nearest marker (Supplementary Material Table 1S).

### Phenotypic variation

Descriptive statistics of percent Karnal bunt infection in both the bi-parent and association mapping populations are presented in Table 1. Across years, the coefficient of variation for percent infection in the bi-parent populations ranged from 89 to 140%, and from 56 to 88% in the GWAS panel. Thus, the inbred lines showed more diverse response to *T*. *indica* infection than the wheat varieties in the association mapping panel. The average percentage infection in the GWAS panel was 22.4%, compared to the average of 13.5–14.1% in the bi-parent populations, because a higher proportion of the inbred lines in the bi-parent populations were resistant to infection (% infection ≤ 5.0).

**Table 1:** Descriptive statistics for percent Karnal bunt infection measured during multiple years of screening for resistance in bi-parent and association mapping populations of common wheat

Experiment/year	Location	Year	Mean KB infection (%)	SE Mean	Std. Dev.	Coef. Var. (%)	Min. (%)	Max. (%)	Skewness
WH542 x HD29									
KB01M	Ludhiana, India	2001	15.00	1.92	20.05	134	0.00	94.00	1.74
KB02M	Ludhiana, India	2002	13.20	1.77	18.48	140	0.00	87.00	1.86
KB03M	Ludhiana, India	2003	15.80	1.73	18.01	114	0.00	73.00	1.25
KB04M	Ludhiana, India	2004	17.43	1.66	17.31	99	0.00	66.00	1.00
KB05M	Ludhiana, India	2005	10.07	1.04	10.88	108	0.00	54.00	1.70
KB15M	Obregon, Mexico	2015	17.28	1.50	15.69	91	0.00	59.00	0.97
KB16M	Obregon, Mexico	2016	10.17	0.99	10.37	102	0.00	44.00	1.54
Mean			14.14	0.59	16.40	116	0.00	94.00	1.59
WH542 x W485									
KB01M	Ludhiana, India	2001	12.93	1.52	16.36	126	0.00	62.00	1.37
KB02M	Ludhiana, India	2002	14.51	1.54	16.55	114	0.00	64.00	1.43
KB03M	Ludhiana, India	2003	14.28	1.31	14.16	99	0.00	68.00	1.31
KB04M	Ludhiana, India	2004	12.89	1.21	13.05	101	0.00	59.00	1.30
KB05M	Ludhiana, India	2005	11.86	1.16	12.45	105	0.00	51.00	1.28
KB15M	Obregon, Mexico	2015	17.88	1.48	15.93	89	0.00	54.83	0.73
KB16M	Obregon, Mexico	2016	10.07	0.98	10.51	104	0.00	50.80	1.76
Mean			13.49	0.51	14.43	107	0.00	68.00	1.33
Association mapping panel									
KB14M	Obregon, Mexico	2014	15.91	1.28	13.94	88	0.00	56.88	0.97
KB15M	Obregon, Mexico	2015	23.61	1.34	14.66	62	0.58	60.44	0.64
KB16M	Obregon, Mexico	2016	27.73	1.42	15.47	56	0.35	63.70	0.32
Mean			22.41	0.82	15.46	69	0.00	63.70	0.58

**Figure 1:**
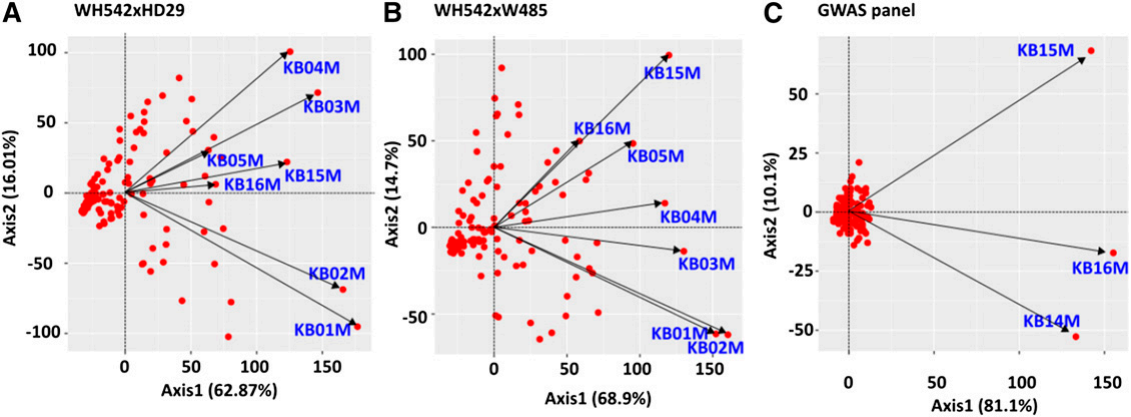
Biplots showing the relationship between data sets for Karnal bunt resistance in (A) WH542×HD29, (B) WH542×W485, and (C) GWAS populations. Axis1 and Axis2 refer to principal components 1 and 2 respectively. The genotypes are represented as red-filled circles. Test environments KB01M, KB02M, KB03M, KB04M, and KB05M were years of experimental evaluations at Punjab Agricultural University, Ludhiana India, while KB14M, KB15M, and KB16M were experiments conducted at CIMMYT station in Obregon, Mexico.

The biplot displays on Figure 1 showed the relationships among years, genotypes and the genotype-by-year interactions (G×E interaction). The first two principal component scores captured between 79% and 91% of the phenotypic variances, and revealed close correlations among the data sets. Consistent with the high percentage infections reported in Table 1, all the test environments had positive PC1 scores, and the relative length of their respective vectors indicated they were equally informative for characterizing Karnal bunt resistance. The angles between the environment vectors were acute (less than 90°), indicating positive correlations between consecutive data sets. This is noteworthy, as it demonstrates a good degree of data reproducibility, even though the data were collected from diverse environments (India and Mexico), and across multiple years. In the bi-parent populations, the minimum correlation coefficient between any two of the environments was 0.39 (*P* < 0.001), and that was between the most discordant data sets, KB01M and KB04M in WH542×HD29 population, and KB01M and KB16M in WH542×W485 cross. Similarly, the minimum correlation coefficient was 0.68 (*P* < 0.001) in the GWAS panel.

In general, the genotypes showed a stable reaction to *T*. *indica* infection, with majority of the lines clustering close to the origin in the biplots (Figure 1). In the GWAS panel, the PC1 axis explained 81% of the phenotypic variation, and this allowed the genotypes to be grouped into those with consistently resistant (negative PC1) and susceptible phenotypes (positive PC1). The genotypes with consistently resistant phenotypes include the Australian varieties, Batavia (syn. QT-4097), Pelsart (syn. QT-4639), RAC-655 (syn. CO-2686-512) and Vulcan (syn. DK-2139), while Halberd (syn. RAC-687), EGA-Bullaring (syn. 93-X-370-M-9-12) and Gladius (syn. RAC-1262) were consistently susceptible to Karnal bunt infection (Supplementary File 1S).

Broad-sense Heritability for Karnal bunt resistance, estimated as the ratio of genetic variance to total phenotypic variance, was 0.52 in the WH542×HD29 population, and 0.61 in the WH542×W485 cross. Genomic heritability, which is the proportion of phenotypic variance that was explained by all genetic markers, was 0.49 for the WH542×HD29 population and 0.40 for the WH542×W485 population. In the GWAS panel, the broad-sense heritability estimate was as 0.71, and the marker-based estimation was 0.61. Taken together, these estimates suggest a high degree of genetic determination for Karnal bunt resistance in the populations under study.

### QTL in the WH542×HD29 population

**Figure 2:**
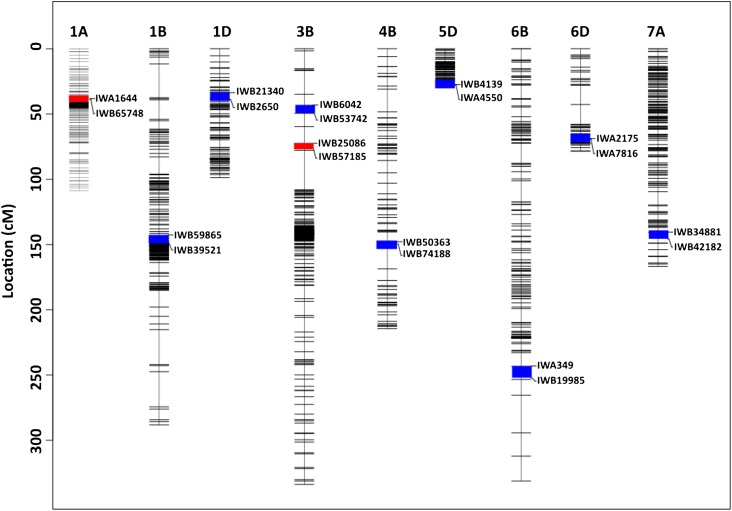
Multi-environment QTL affecting Karnal bunt resistance in WH542×HD29 (red bar) and WH542×W485 (blue bar) RIL populations. The QTL are projected onto a consensus map constructed for both populations. Chromosomes harboring the QTL are represented by vertical lines, and each horizontal black line represents one of the unique SNP markers. The genetic distances are reported on the scales to the left of the chromosomes.

Two main-effect QTL were detected in the WH542×HD29 population. The major locus (R^2^ = 27.8%) was detected on chromosome 3B, at the interval between IWB25086 and IWB57185 (Table 2; Figure 2). The closest SNP marker was IWB57185, and the allele for resistance (Supplementary Material Figure 2S) was inherited from the resistant parent, HD29. The effect of the allele on Karnal bunt resistance was consistent across environments (Supplementary Material Figure 2S). The second locus was mapped to chromosomes 1A (Figure 2), and explained 12.2% of the phenotypic variance. Linear contrast between the ‘A’ and ‘C’ alleles at the chromosome 1A locus (IWA1644) showed that it was the ‘C’ allele that conferred Karnal bunt resistance (Supplementary Material Figure 2S). The allele was inherited from the susceptible parent, WH542, and an ANOVA test for marker-by-environment interaction was not significant (*P* = 0.19).

**Table 2:** Summary of QTL identified in bi-parent and GWAS populations of common wheat

Closest SNP marker	Chrom.	Contig ID	Physical location (Mb)	*P* value	Size*^a^*	Parent with fav. allele	Expl. Var. (%)
WH542×HD29 RIL population							
IWA1644	1A	1AL_3934771	590.0	0.000	4.08	WH542	12.2
IWB57185	3B	3B_10665467	16.5	0.000	−6.17	HD29	27.8
WH542×W485 RIL population							
IWB59865	1B	1BS_3415168	17.7	0.000	−5.39	W485	15.8
IWB2650	1D	1DS_1878443	10.4	0.000	6.59	WH542	23.6
IWB53742	3B	3B_10760567	45.3	0.000	3.43	WH542	6.4
IWB74188	4B	4BL_6962777	651.8	0.000	−3.49	W485	6.6
IWA4550	5D	5DL_4512777	192.3	0.000	−2.88	W485	4.5
IWA349	6B	6BL_4368768	688.2	0.000	−2.85	W485	4.4
IWA7816	6D	6DL_518479	398.2	0.000	3.33	WH542	6.0
IWB34881	7A	7AL_4556858	634.4	0.000	3.72	WH542	7.5
Genome-wide association panel							
IWA7633	2B	2BS_5175914	6.3	0.000	−4.51	RAC655	2.8
IWA5637	2D	2DL_9872584	369.5	0.000	−4.70	RAC655	2.4
IWA2008	3A	3AL_4451937	689.5	0.000	−5.96	RAC655	2.7
IWA3835	3B	3B_10756975	664.9	0.000	−8.47	Batavia	10.1
IWA1123	3D	3DS_2593299	33.2	0.000	−7.80	Batavia	3.9
IWA2087	4B	4BL_6970737	670.4	0.000	−3.56	RAC655	1.3
IWA7302	5A	5AS_1502365	19.2	0.000	−8.87	RAC655	6.8

a Effect size in percentage Karnal bunt infection.

**Figure 3:**
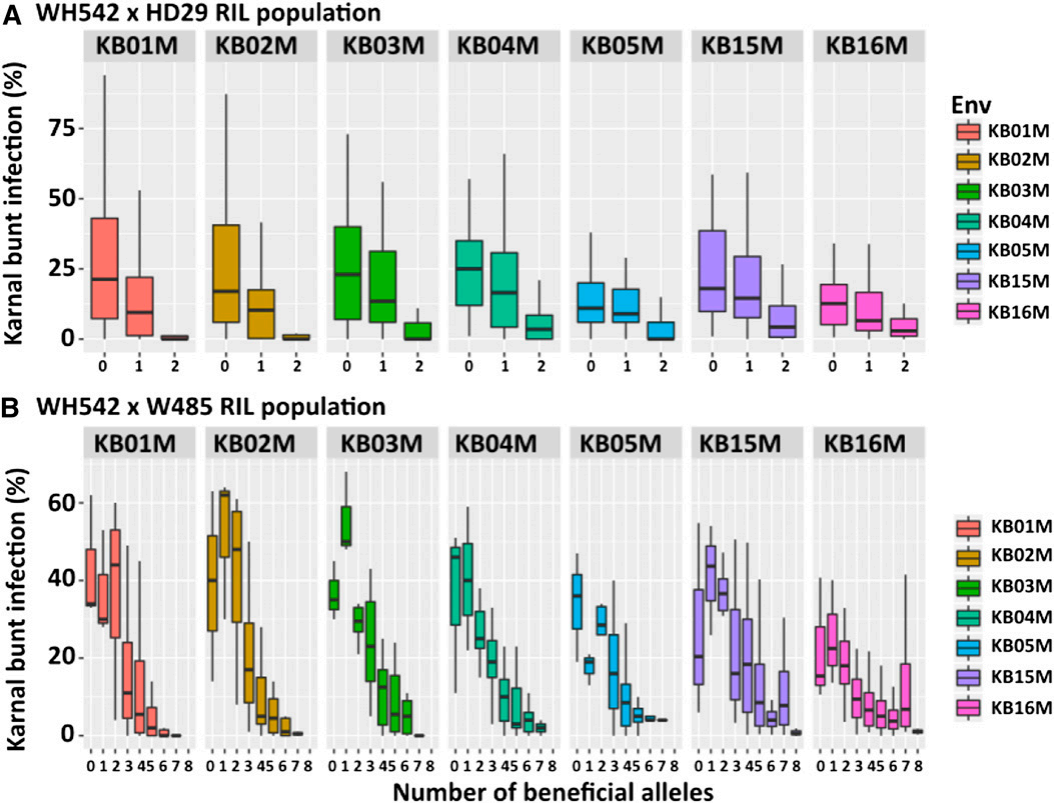
Effects of beneficial alleles on Karnal bunt resistance in (a) WH542×HD29, and (b) WH542×W485 populations. The environments (KB01M, KB02M, KB03M, KB04M, KB05M, KB15M, and KB16M) were years of experimental evaluations at Punjab Agricultural University, Ludhiana India, and Obregon, Mexico.

There was no significant interaction between the QTL on chromosome 1A and 3B (*P* = 0.35), indicating that the two loci exhibited partial dominance. A chi-square test confirmed incomplete dominance of the alleles, with the haplotype segregation ratio of 1:2:1 (Figure 3) not significantly different from the expected (Chi-squared = 2.23; p-value = 0.33). Across environments, genotypes carrying two of the beneficial alleles averaged 5.1% Karnal bunt infection, compared to 15.8% for those with either of the dominant alleles, and 23.0% for those with none of the alleles (Figure 3).

### QTL in the WH542×W485 population

In the WH542×W485 population, eight QTL were detected (Table 2; Figure 2), explaining between 4% and 24% of the genetic variance for Karnal bunt resistance (Table 1). The largest effect was detected on chromosome 1D, which explained 23.6% of the genetic variance. The QTL mapped to the short arm of chromosome 1D, closest to the SNP marker, IWB2650, with the allele for resistance (Supplementary Material Figure 3S) inherited from the susceptible parent. The allele for resistance on chromosomes 3B, 6D and 7A were also inherited from WH542, but the resistance alleles at chromosomes 1B, 4B, 5D and 6B were contributed by the resistant parent, W485 (Table 1). All the QTL exhibited stable expressions across environments, except the locus on chromosome 1B (IWB59865) (Supplementary Material Figure 3S). Most of the identified QTL showed significant (*P* ≤ 0.05) digenic epistatic interactions. Haplotype analysis of beneficial alleles showed that a combination of the eight detected QTL was necessary to achieve complete resistance (zero Karnal bunt infection) (Figure 3).

### QTL detection in association mapping panel

**Figure 4:**
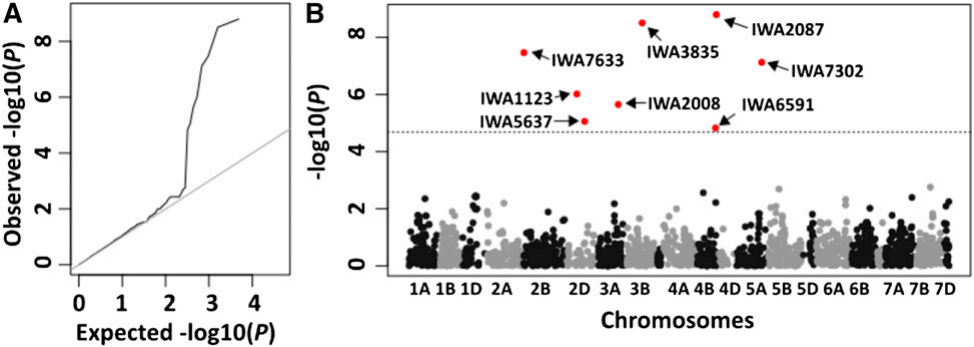
Genome-wide scan for SNP markers associated with Karnal bunt resistance in an association mapping panel of commercial wheat varieties. The plots show: (a) QQplot of the observed *P*-value distribution, and (b) Manhattan plot of the identified loci above the significance threshold (broken line), with the chromosomes on the X-axis and the genome-wide scan –log10(*P*-values) on the Yaxis.

The MLMM analysis detected eight significant (–log10 (*P*) ≥ 4.68) markers, after accounting for population structure and genetic relatedness (Figure 4). The most significant *P*-values were associated with SNP markers on chromosomes 3B and 4B (Table 1). Significant loci were also detected on the short arm of chromosome 2B (2BS_5175914), the long arm of chromosome 2D (2DL_9872584), and the long arm of chromosome 3A (3AL_4451937). The other significant regions include the short arm of chromosome 3D and the short arm of chromosome 5A (Table 1). When haplotypes were considered, varieties carrying the most favorable combination averaged 4.1% in Karnal bunt infection, compared with an average of 51.7% in the group carrying the least favorable haplotype.

### Meta-QTL for KB resistance

**Figure 5:**
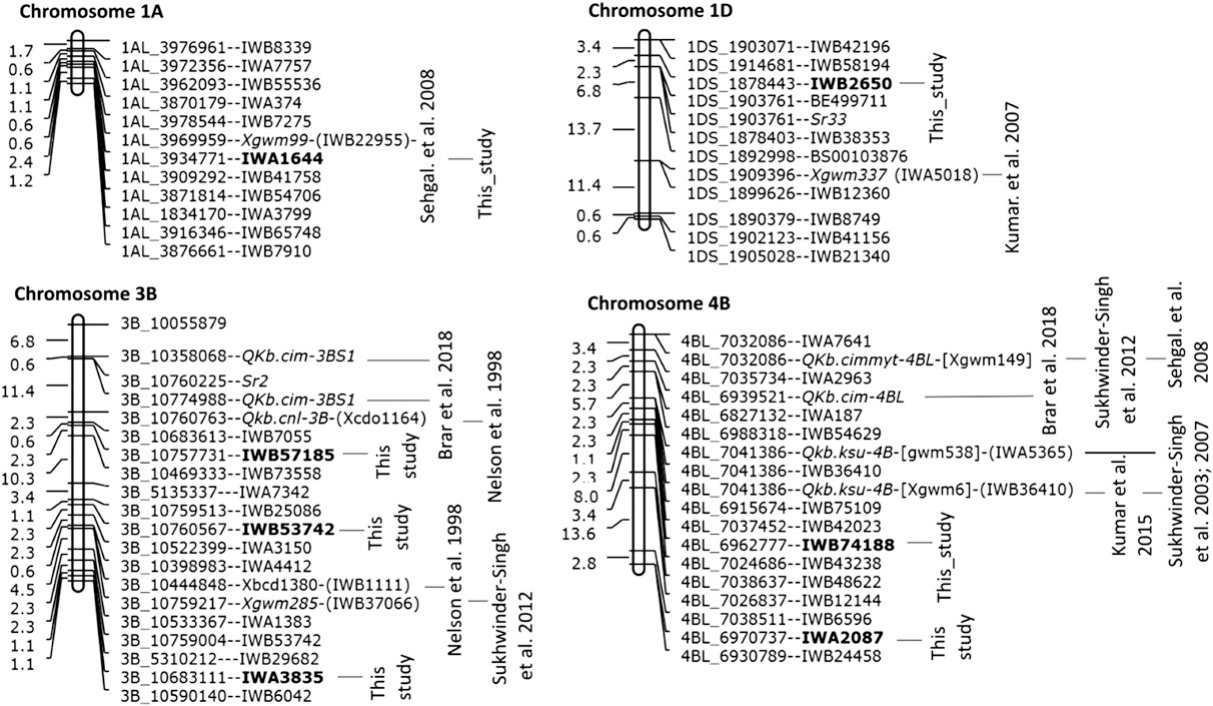
Meta-QTL regions associated with Karnal bunt resistance from independent studies. Markers identified from different studies were projected onto the POPSEQ genetic map (Chapman *et al.* 2015).

Meta-QTL analysis identified four genomic regions where results from this and previous studies coalesced (Figure 5). The largest clusters were detected on chromosomes 3B and 4B. The region on chromosome 3B contained two non-overlapping meta-QTL within a 28.8 Mb interval. The first showed the clustering of QTL identified in the WH542×HD29 population with *Qkb.cnl-3B* reported by Nelson *et al.* 1998, and the more recent locus, *Qkb.cim-3BS* identified by Brar *et al.* (2018); the other cluster was the co-location between QTL detected in the WH542×W485 population (IWB53742) and GWAS panel (IWA3835) with the Xgwm285 SSR and Xbcd1380 loci reported by Sukhwinder-Singh *et al.* (2012) and Nelson *et al.* 1998, respectively (Figure 5).

The meta-QTL cluster on chromosome 4B involved QTL results from seven independent studies (Figure 5). These include the QTL detected in this study from the WH542×W485 population (IWB74188) and the GWAS panel (IWA2087), along with previously detected loci, *Qkb.ksu-4B*, *QKb.cimmyt-4BL*, *Qkb.cim-4BL*, and the markers detected by Kumar *et al.* (2015) and Sehgal *et al.* (2008), within an interval of 49.5 cM.

The third meta-QTL was located on chromosome 1A, where the QTL detected in this study overlapped with the locus reported by Sehgal *et al.* (2008). The fourth cluster was found on the short arm of chromosome 1D (Figure 5), where the QTL detected in this study co-located with the locus identified by Kumar *et al.* (2007).

## Discussion

Resistance to Karnal bunt infection is one of the most difficult traits to phenotype in the field. The method of artificial inoculation by manual injection of sporidia into the boot is time-consuming and labor-intensive, and the disease incidence is highly dependent on environmental conditions (Sukhwinder-Singh *et al.* 2003; Singh *et al.* 2007; Brooks *et al.* 2006). Knowledge of the genomic architecture underlying the resistance will provide tools for breeders to efficiently track the resistance genes during introgression in cultivar development. Both broad-sense and genomic heritability estimates indicated a high degree of genetic determination for Karnal bunt resistance in the populations used for this study. A recent study by Brar *et al.* (2018) reported higher heritability estimates of 0.75 and 0.78 in two wheat populations. These findings suggest that Karnal bunt resistance in common wheat is highly amenable to QTL mapping. The high heritability estimates may be due to the phenotyping protocols established by Fuentes-Davila *et al.* (1995) that limits environmental or unexplained variations during the field disease nursery experiments.

The current study provides clarification of quantitative basis for Karnal bunt resistance in wheat, for which additive and additive × additive gene action is reported to be predominant. Across populations and years, the phenotypic distribution for percent infection was positively skewed toward Karnal bunt resistance (Table 1), indicating presence of major-effect genes or genes with epistatic effects. The study has identified major and minor QTL controlling Karnal bunt resistance in different bi-parent mapping populations, with resistance alleles contributed by both parents. Rajarammohan *et al.* (2017) are of the view that when major QTL are detected in different populations, it suggests that different genes/mechanisms may be activated in different accessions in response to the same pathogen. This will agree with reports that different genes control Karnal bunt resistance in different wheat accessions (*e.g.*, the report of Fuentes-Davila *et al.* 1995).

The identified QTL were largely population-specific and showed significant (*P* ≤ 0.05) digenic epistatic interactions, such that no single QTL provided complete resistance and the presence of beneficial alleles were necessary to achieve near-zero Karnal bunt infection (Figure 3). This is consistent with reports that suggest epistatic variance might be an integral part of inheritance for Karnal bunt resistance in common wheat (Sharma *et al.* 1995; Nagarajan *et al.* 1997). In a study of Mexican bread-wheat genotypes, Singh *et al.* (1995) reported that the genotypes with two genes for resistance expressed a higher level of resistance than those with a single gene and, therefore, are better sources of resistance to Karnal bunt. Similarly, Fuentes-Davila *et al.* (1995), who reported six different genes in six resistant wheat accessions, concluded that the accumulation of all genes in a single genotype will be required in order to confer high levels of resistance.

There is a measure of ambiguity regarding the number of genes carried by resistant stocks. Fuentes-Davila *et al.* (1995) reported six different genes were present in six different resistant wheat accessions, and according to Sharma *et al.* (2005), there may be as many as nine genes governing Karnal bunt resistance in the four parents. Sharma *et al.* (2005) studied Karnal bunt resistance in the cross of WH542×HD29 and WH542×W485, and from the phenotypic segregation ratios, the authors concluded that the resistant parents carried two major genes for resistance. Another study of WH542×HD29 RIL population by Kumar *et al.* (2007), however, suggested that WH542 might carry a minor gene for Karnal bunt resistance. Kumar *et al.* (2007)’s conclusions were based on presence of transgressive segregants, but in the current study, disease resistance alleles were found to be contributed by both resistant and susceptible parents in the two crosses. A similar result was recently reported by Brar *et al.* (2018), thus indicating that apparently disease susceptible parents may possess alleles that confer disease resistance. In soybean, Hnetkovsky *et al.* (1996) found two QTL for sudden death syndrome, with beneficial alleles derived from the resistant and susceptible parents.

There is no definitive chromosomal assignment of genes controlling Karnal bunt resistance in common wheat (Nelson *et al.* 1998), although Gill *et al.* (1983) suggested that chromosomes 1A and 1D might harbor a complementary gene system for resistance, and that chromosome 4D possessed a dominant gene controlling susceptibility. According to Nagarajan *et al.* (1997), a monosomic analysis using three resistant lines HD29, WL6975 and WL2348 and the susceptible WL711 showed that the genes governing resistance to Karnal bunt are located on chromosomes 1D, 2D, 3B, 3D, 5B and 7A. We used the method of meta-QTL analysis to summarize previously reported QTL in order to identify unique chromosomes with QTL clusters that underpin Karnal bunt resistance. Four clusters were identified on chromosome 1A, 1D, 3B and 4B, with the largest clusters on chromosomes 3B and 4B (Figure 5). The chromosomes 1A and 1D clusters are essentially verifications of the loci identified in this current study. The chromosome 1A region was first reported by Sehgal *et al.* (2008) in a set of isogenic lines, and the Chromosome 1D locus was also first reported by Kumar *et al.* (2007) in the cross of WH542×HD29 and later verified in the cross of H567.71 (resistant) × WH542 (susceptible) by Kumar *et al.* (2015).

The results provide definitive chromosomal assignments for QTL/genes controlling Karnal bunt resistance in common wheat, which will be useful in pre-emptive breeding in wheat-producing areas where quarantine regulations prohibits phenotyping in the field. The chromosome 3B and 4B clusters represent QTL identified in five to seven independent studies. The chromosome 4B region was originally identified in the cross of WL711×HD29 (Sukhwinder-Singh *et al.* 2003), and verified by Brooks *et al.* (2006). In this study, it was not detected in the WH542×HD29 population, confirming the lack of its segregation in the population, as reported by Singh *et al.* (2007). However, it was detected in the WH542×W485 cross (Table 1), in the cross of H567.71×WH542 (Kumar *et al.* 2015), and in a set of Karnal bunt-resistant near-isogenic lines developed from the cross PBW343×KBRL22 (Sehgal *et al.* 2008). The regions on chromosome 3B and 4B may therefore represent chromosomal regions for genes controlling Karnal bunt resistance in common wheat.

Results from genetic linkage and association mapping are often not identical (Lou *et al.* 2015; Albert *et al.* 2016; Li *et al.* 2016), but they are complementary, that is, results from genome-wide association studies often confirm previously detected QTL (Waugh *et al.* 2010; Raman *et al.* 2010; Mulki *et al.* 2012; Emebiri *et al.* 2017). In the current study, the results from GWA mapping validated the consensus regions on chromosomes 3B and 4B identified from the bi-parent populations (Figure 5), but also identified novel loci for Karnal bunt resistance. Quantitative disease resistance entails activation of genes that drive the innate immune system of a plant and also those that encode other defense-related outputs (Corwin and Kliebenstein 2017). The majority of cloned disease resistance genes in wheat function by pathogen recognition, activated by genes that encode leucine-rich repeat family proteins (Keller *et al.* 2018). In this study, the GWA mapping revealed a set of novel loci on chromosome 2B and 3DL that were not detected in the bi-parent populations. The detection of novel disease-resistance loci from GWA mapping is one of the advantages of GWA mapping, that is, it exploits a wider genetic variation than is present in bi-parent populations. The results indicate that genetic variation at an expanded number of loci may underpin the quantitative nature of resistance to Karnal bunt in common wheat.

### Implications for breeding

Resistance to Karnal bunt infection has been found mostly in genotypes that are of poor agronomic value, such as the wild wheat relatives (Chhuneja *et al.* 2008), synthetic genotypes (Mujeeb-Kazi *et al.* 2006), and wheat landaces (Fuentes-Davila and Rajaram 1994; Villareal *et al.* 1995). The markers identified in this study would provide efficient tools for characterizing these sources of resistance, and the possible origin of the genes they carry. For this purpose, the identification of candidate genes and development of gene-based diagnostic markers will be of paramount importance. The meta-QTL shown in Figure 5 provide starting points for targeted gene discovery using the genomic resources now available at the International Wheat Genome Sequencing Consortium (IWGSC) database.

The results from this study reinforce the view that complete resistance to Karnal bunt infection would require inheritance of favorable alleles at multiple gene loci (Singh *et al.* 1995; Sharma *et al.* 2004). Pyramiding or stacking such alleles into a single cultivar can be achieved by using the identified molecular markers to track the resistance genes during the various stages of backcrossing. This will be especially useful for pre-emptive breeding, since the method of artificial inoculation and phenotypic selection cannot be carried out in wheat-exporting countries that are free of the disease. However, the role of epistatic interactions in Karnal bunt resistance will need to be properly defined, because interactions among QTL/genes and environmental factors can make substantial contributions to variation in complex traits such as disease susceptibility (Carlborg and Haley 2004). In fact, most published data suggest that marker-assisted selection may not be successful for complex, multigenic traits that involve epistasis (Langridge and Waugh 2019). New and innovative strategies will be required, such as the method of genomic selection (Meuwissen *et al.* 2001), which uses dense genome-wide markers to predict breeding value of individuals. In the best-case scenario, breeders can select the best performing genotypes from the population for use in their crossing block, without the need to phenotype the plants themselves. The decisions will depend on accuracy of the predictions, but these have not been investigated for Karnal bunt resistance in common wheat.

### Summary and conclusions

Karnal bunt resistance in common wheat is a complex trait, with reports of different genes controlling the resistance in different accessions. The current study used multi-environment experiments of two bi-parent RIL populations, and a GWAS panel of commercial varieties to unravel the genetic basis for resistance. High heritability estimates obtained for the populations provided a good impetus for QTL analysis, and the results showed different major and minor QTL in the two bi-parent mapping populations, with resistance alleles contributed by both parents. The detection of major QTL in different populations supports the view that different wheat accessions carry different genes for Karnal bunt resistance. The GWA mapping validated consensus QTL regions on chromosomes 3B and 4B, but also identified novel loci not previously associated with Karnal bunt resistance. The identified SNP markers in close proximity to the QTL will provide breeder-friendly resources to accelerate transfer of resistance from parental genetic stocks into commercial wheat cultivars.
